# Prediction of protein-protein interaction types using association rule based classification

**DOI:** 10.1186/1471-2105-10-36

**Published:** 2009-01-28

**Authors:** Sung Hee Park, José A Reyes, David R Gilbert, Ji Woong Kim, Sangsoo Kim

**Affiliations:** 1 Department of Bioinformatics & Life Science, Soongsil University, Seoul, 156-743, Korea; 2 School of Information Systems, Computing and Mathematics, Brunel University, Uxbridge, UB8 3PH, UK; 3 Facultad de Ingeniería, Universidad de Talca, Talca, Chile; 4 Equispharm Co., Ltd, Seoul, 443-766, Korea

## Abstract

**Background:**

Protein-protein interactions (PPI) can be classified according to their characteristics into, for example obligate or transient interactions. The identification and characterization of these PPI types may help in the functional annotation of new protein complexes and in the prediction of protein interaction partners by knowledge driven approaches.

**Results:**

This work addresses pattern discovery of the interaction sites for four different interaction types to characterize and uses them for the prediction of PPI types employing Association Rule Based Classification (ARBC) which includes association rule generation and posterior classification. We incorporated domain information from protein complexes in SCOP proteins and identified 354 domain-interaction sites. 14 interface properties were calculated from amino acid and secondary structure composition and then used to generate a set of association rules characterizing these domain-interaction sites employing the APRIORI algorithm. Our results regarding the classification of PPI types based on a set of discovered association rules shows that the discriminative ability of association rules can significantly impact on the prediction power of classification models. We also showed that the accuracy of the classification can be improved through the use of structural domain information and also the use of secondary structure content.

**Conclusion:**

The advantage of our approach is that we can extract biologically significant information from the interpretation of the discovered association rules in terms of understandability and interpretability of rules. A web application based on our method can be found at

## Background

Protein-Protein Interactions (PPIs) play a key role in many essential biological processes in cells, including signal transduction, transport, cellular motion and gene regulation. The comprehensive analysis of these biological interactions has been regarded as very significant for the understanding of underlying mechanisms involved in cellular processes.

Computational approaches for the prediction of PPI based on atomic level interactions can accurately determine the binding affinity and the specificity of binding partners. Thus, structure based prediction methods including modeling of PPI by homology modeling, threading-based methods and protein-protein docking are more accurate than methods that do not employ structure data. A major drawback of these structure-based methods is the relatively low coverage of available crystallized protein complexes in the Protein Data Bank (PDB) [1]. This is especially the case for those proteins associated with transient interactions, which is the majority of functional PPIs, and these do not form complexes stable enough for x-ray crystallography [2]. Due to these restrictions the detailed analysis of the structure of protein complexes, specifically the area related to the interaction site between proteins, can reveal important clues for the understanding of protein functions and also characterize the specificity of these interaction regions.

The prediction of protein interaction sites has gained much attention in recent years with over 20 different methods proposed [3]. Interaction regions can be characterized by a diverse set of physico-chemical properties [4-6], topological properties [7] and conserved residues [8]. A variety of studies have employed different classification approaches including Support Vector Machines [9-12], Random Forests [13] and Neural Networks [14]. These studies have shown that the interfaces of interaction sites share common properties that distinguish them from the rest of the protein [15,16,4]. Despite their good performance in the prediction of protein interaction sites, these machine learning approaches generate final prediction models which do not provide users with explicit rules and thus result in low interpretability of the results and poor knowledge extraction capability.

The identification, analysis and characterization of different PPI types can be classified according to their life time and binding affinity into four main classes [16,17,11]: obligate permanent interactions involving homo or hetero obligomers and non-obligate transient interactions involving Enzyme-inhibitor or non Enzyme-inhibitor. In obligate protein interactions, protomers which are not individually structurally stable in vivo, form permanent functional complexes that are stable and exist in their complexed form. Protomers of non-obligate interactions are independently stable and can form transient or permanent complexes. Non Enzyme-inhibitors are participants in transient interactions not involving enzymes and their protein inhibitors.

The characterization of PPI types can help for instance in the functional annotation of newly crystallized protein complexes as suggested in [17]. Several studies have been developed in this direction, focused on the discrimination of different PPI types with the aim of characterizing transient and obligate protein complexes [17,18]. These include the statistical analysis of the interface properties [19], and the analysis from an evolutionary view of issues related to these interactions [20].

A recent computational approach [12] classified binary protein complexes into three categories (obligate interactions, non-obligate interactions and crystal packing) using six interface properties and employing Support Vector Machines (SVM). These studies [17-19,12] have improved our overall understanding of the differences between obligatory and non-obligatory or between permanent and transient interactions. Their analysis methods based on correlation drawn by the 2Ds plot of two properties has shown that a single property does not completely distinguish the different PPI types of interaction sites and the combination of more properties can give more strength to the discrimination of PPI types.

In our work we describe a computational approach for the prediction of PPI types employing association rule based classification (ARBC) [21,22], which includes association rule generation and posterior classification based on the discovered rules. In a similar manner to previous approaches we investigate diverse properties associated with the interface of protein complexes. But instead of considering the entire interface area between two proteins we only consider the region associated with domain information by using the SCOP classification [23]. The use of domain profile pairs can provide better prediction of protein interactions than the use of full-length protein sequences as reported in Wojcik *et al*. [24]. In addition we also incorporate secondary structure information related to these domain-binding sites into our predictive approach. These features appear to be useful for the characterization and classification of binding interfaces as reported recently in Guharoy *et al*. [25].

The main aim of this work is to discover patterns, in the form of association rules, that characterize interaction sites in different PPI types. An important advantage of using such a classification approach is the interpretability of the final predictive model based on the analysis of the discovered set of rules. We give a detailed interpretation of discovered association rules in order to find common and specific patterns which are biologically significant and can be used to distinguish one PPI type from others. Here we focus on the prediction of four different PPI types (i.e. transient Enzyme inhibitor/Non Enzyme inhibitor and permanent homo/hetero obligomers), trying to gain more specific insights into the characterization of diverse kinds of interactions.

## Methods

### Interaction Data

We employed the same data set of non-redundant interacting protein complexes reported by [11]. The set of 147 complexes was selected from a comprehensive set of 180 proteins taken from the PDB. 25 of these 147 complexes are involved in Enzyme-inhibitor (ENZ) interactions, 21 in non-Enzyme-inhibitor (nonENZ) interactions, 14 in hetero-obligate (HET) interaction, and 87 in homo-obligate (HOM) interactions as shown in Table 1. Proteins sharing > 20% sequence identity with a higher resolution structure of the same complex type were removed. Crystal packing structures were also eliminated by investigating the evidence in the literature that the complex occurs naturally and is stable as a dimer. NMR structures were not used, neither were mutant complexes nor structures whose resolution was > 3.0 Å; permanent complexes are more easily available from stable complexes by x-ray crystallography. Transient PPIs often neither form stable complexes nor give good NMR structures. This is reflected in the small number of validated transient complexes available in the PDB.

**Table 1:** Data set of protein complexes

Type Name	Type of Interaction	#. of Complexes	#. of Domains
ENZ^*a*^	Enzyme-inhibitors	25	49
nonEnz^*b*^	Non Enzyme-inhibitors	21	47
HET^*c*^	Hetero-obligomers	14	33
HOM^*d*^	Homo-obligomers	87	225

**Total**		**147**	**354**

^*a*^*EN Z*: Enzyme-inhibitor interactions;

^*b*^*nonENZ*: non-Enzyme-inhibitor interactions;

^*c*^*HET*: Hetero-obligate interactions;

^*d*^*HOM*: Homo-obligate interactions.

### Definition of interface and dom-face

An *interface *is a set of interacting atoms whose Solvent Accessible Surface Area (SASA) is decreased by > 1 *Å*^2 ^upon the formation of a complex [4]. The SASA for each atom was calculated using MSMS [26] with a probe sphere of radius 1.5 Å. Given a pair of interacting proteins, we define a set of interacting atoms for a single protomer as a *face*. An *interface *comprises a pair of interacting *faces*. We define the set of atoms comprising the *face *of a single domain as a *dom-face*. In order to calculate *dom-faces*, the interfaces extracted from complexes are mapped onto ranges of SCOP 1.65 domain definitions [23]. A total of 354 SCOP domains were extracted related to form the 147 protein complexes considered in our study of the different PPI types, see Table 1.

### Description of dom-face

We generated 14 different physico-chemical properties and structural features to characterize each of the *dom-faces *considered in our study including: *dom-face *area (df-ASA), hydrophobicity (HH), residue propensity (inPro), number of amino acids (nAA), number of atoms (nAtom), number of Secondary Structure Elements (nSSE), length of consecutive residues (LCS), number of fragments (nFrag), Size ratio of dom-face area to domain area (sRatio), Secondary Structure Elements (SSEs) content (Helix, Strand, Non-Regular) and SCOP class number (SCOPClass). Hydrophobicity and residue propensity were analyzed in the same way as Jones and Thornton [4].

The solvent accessible surface area (SASA) of a *dom-face *is calculated as the sum of the total decreased SASA for the interface atoms in a domain, see Equation 1. If A and B are two protomers in the complex AB, *SASA*_*A*_, *SASA*_*B *_and *SASA*_*AB *_are SASA values for A, B, and AB respectively, and *n *is the total number of interface atoms in a domain presented in protomers A and B, then

(1) $$dom\text{-}face\;Area=(\sum_{i=1}^{n}\left(SASA_{A}(i),SASA_{B}(i)),-SASA_{AB}(i)\right)$$

We employed the hydrophobicity scale of Fauchere and Pliska [27] to estimate the average hydrophobicity value for each *dom-face*. The average hydrophobicity (HH) is calculated using Equation 2, where *HI*_*AA *_is the hydrophobicity value for each amino acid residue and *N*_*AA *_is the number of residues in a *dom-face*.

(2) $$HH=\frac{\sum_{i=1}^{l}HI_{AA}}{N_{AA}}$$

Residue propensity (inPro) indicates the relative frequency of different amino acid (AA) residues in *dom-faces *of complexes. We estimated residue propensities for all *dom-faces *using Equation 3 [16], where *AAP*_*i *_in Supplementary Table One [see Additional file 1] is the natural logarithm of each AA propensity and *N*_*R *_is the total number of residues in a *dom-face*. AA propensities for 20 amino acids over our data sets of 354 dom-faces were calculated using Supplementary Equation One [see Additional file 1].

(3) $$inPro=\frac{\sum_{i=1}^{n}AAP_{i}}{N_{R}}$$

In order to analyze the size of interaction sites we computed the ratio between *dom-face *and the whole domain area (SR) employing Equation 4.

(4) $$SR=\frac{ASA_{dom\text{-}face}}{ASA_{domain}}$$

The sequence continuity in the interaction sites is described by calculating average length (number) of consecutive residues (LCS) and counting the number of consecutive residues (nFrag) in *dom-faces*. The SSE content is calculated by the percentages of interaction atoms located in Secondary Structure Elements (SSEs), classified using the types defined in DSSP [28]: helix, strand and non regular regions (turn, bend and loop). PPI types become the heads of association rules in ARM and the target classes in our classification. We used four different types of PPI, namely Enzyme inhibitor/Non Enzyme inhibitor as transient interaction types and homo/hetero obligomers as permanent interaction types. Other properties estimated for the diverse *dom-face*s analyzed were the SCOP class number at the first level of the SCOP hierarchy, the number of AA, the number of atoms and the number of SSEs present in the different interaction interfaces.

### Association Rule Based Classification

The problem of predicting PPI types for a given complex of binary proteins is transformed into the task of assigning a pre-determined target class (i.e., homo/hetero obligate and non-obligate) using properties of interaction sites. We applied an efficient association rules based classification method (ARBC) to perform classification based on rules generated by Association Rule Mining (ARM). Previous studies [21,22] have proposed that ARBC consistently outperforms other rule-based classifiers such as decision trees. ARBC comprises three main steps: association rule generation, pruning association rules and classification based on association rules.

#### Association rule generation

In our approach we employed Association Rule Mining to discover a set of frequent patterns expressed as association rules describing the relationship between properties of PPI interaction sites and PPI types. Association rules have the form *R*: *X *→ *Y *[*c*, *s*], where *X *and *Y *are the body and the head of the rule respectively. *X *and *Y *are disjoint predicates (*X *∩ *Y *= *ϕ*). Each *X *and *Y *consists of a conjunction of distinct predicates which describe properties related to interaction sites. Note that we can consider a conjunction as a set for our purposes. In our approach, the heads of all rules *Y *are restricted to be one of the PPI types considered which are the target classes defined in this task. The strength of the association rules can be measured in terms of their *support *(*s*) and *confidence *(*c*). The support of a rule (*X *→ *Y*) is the probability that the cases in a database contain both *X *and *Y*. The confidence of the rule is the probability that a case contains *Y *given that it contains *X*.

The generation of association rules was carried out employing the APRIORI algorithm [29]. We used the 10 g Oracle Data Miner (ODM) software which implements the APRIORI algorithm to compute the type of association rules required for our ARBC approach. We set a minimum support and confidence of 3% and 25% respectively to reduce the number of association rules generated. Association mining is not directly applicable to real valued continuous data such as some of the *dom-face *properties we generated. Hence we used discretisation to manipulate continuous attributes before the ARM process was executed. In this process adjacent values of continuous data were binned into a finite number of intervals.

#### Pruning association rules

The number of rules generated by ARM can be very large. It is necessary to prune the set of association rules by removing redundant information in order to make the classification more efficient.

Given two rules *R*_1_: *X*_1 _→ *Y*_1 _and *R*_2_: *X*_2 _→ *Y*_2_, we define:

*Definition 1*. **The significance of a rule**: *R*_1 _is more significant than *R*_2 _if and only if either (1) *conf *(*R*_1_) > *conf *(*R*_2_) or (2) *conf *(*R*_1_) = *conf *(*R*_2_) but *sup*(*R*_1_) > *sup*(*R*_2_) or (3) *R*_1 _has fewer attributes in its left hand side than *R*_2 _◇

*Definition 2*. **General rule**: Given two rules *R*_1_: *X*_1 _→ *Y*_1 _and *R*_2_: *X*_2 _→ *Y*_2_, *R*_1 _is a general rule if and only if *X*_1 _⊆ *X*_2 _◇

*Definition 3*. **Overlapping rule**: Given two rules *R*_1_: *X*_1 _→ *Y*_1 _and *R*_2_: *X*_2 _→ *Y*_2_, then *R*_3_: *X*_1 _∨ *X*_2 _→ *Y*_1_(*conf *(*R*_1_), *sup*(*R*_1_)) ∨ *Y*_2_(*conf *(*R*_2_), *sup*(*R*_2_)) is an overlapping rule if and only if *X*_1 _= *X*_2 _and *Y*_1 _≠ *Y*_2_

◇ If the body of a rule *R*_1 _is identical to the body of a rule *R*_2 _and the head of rule *R*_1 _is inconsistent with that of rule *R*_2_, then an overlapping rule *R*_3 _between two different PPI types can be identified.

Overlapping rules can be considered as common rules between two or more PPI types. On the other hand unique rules are distinctive patterns which can be used to classify interaction sites into different PPI types.

We then evaluated the following condition in order to prune the set of association rules previously generated. Given two rules *R*_1 _and *R*_2_, where *R*_1 _is a general rule w.r.t. *R*_2_, ARBC eliminate *R*_2 _if *R*_1 _has more significance than *R*_2_. Sets of unique and overlapping rules were generated with the pruning procedure used in the classification.

#### Classification

In the classification step we employed the pruned set of unique and overlapping rules to generate a *rule profile *consisting of an *m *× *n *matrix, where *m *is the number of examples (i.e. *dom-faces*) and *n *is the number of different association rules obtained after the pruning step. Each row of this matrix represents one of the *dom-faces *considered in our research and is associated with one of the PPI types we wish to classify. The *rule profile *matrix takes values of 1 or 0 depending whether the different rules are contingent or not on the respective *dom-face *example. A similar approach was previously employed in [30] for protein structure comparison. The *rule profile *matrix was generated following Algorithm 1 and then used as input to the ARBC process.

**Algorithm 1 **Generation of a rule profile

Input:   A set of rules (*R*_1_, ⋯, *R*_*n*_) and

      A set of training data comprising m objects (*O*_1_,⋯, *O*_*m*_)

Output:   An *m *× *n *matrix, *RProfile*(*i*, *j*)(1 ≤ *i *≤ *m *and 1 ≤ *j *≤ *n*)

Method:

1.            Sort rules in the descending order of confidence and support

2.            for each rule *R*_*j *_in the descending order of the rules

     for each data object *O*_*i *_in the training data

          find match between *O*_*i *_and rule *R*_*j*_

               if *match*(*O*_*i*_, *R*_*j*_)

                    set RProfile(*i*, *j*) = 1

               else

                    set RProfile(*i*, *j*) = 0

          end-for

     end-for

We evaluated several classification techniques for this task including Decision Trees (DT), Random Forest (RF), K Nearest Neighbor (KNN), Support Vector Machines (SVM), and Naive Bayes (NB). The WEKA machine learning library [31] was used to perform these experiments. We also performed conventional classification based only on the physicochemical properties of the different *dom-faces *examples, without generating a set of association rules (CWAR). This was done in order to evaluate if the employment of the ARBC approach could be associated with a loss of information of some interacting complexes due, for example, to the pruning step or the discretisation of continuous value feature information. In all cases a 10 fold cross validation procedure was performed. Because the task of classification of different PPI types involves imbalanced classes (see Table 1) we utilized an over-sampling strategy, incrementing the number of instances associated with those PPI types with few examples.

## Results and Discussion

### Analysis of dom-face Properties

We found that 98.8% of the interaction sites studied are contained within corresponding ranges of SCOP domains. This suggests that the analysis of interaction sites based on structural domains (i.e. *dom-face*) does not lose interaction information.

Average values of diverse *dom-face *properties for different PPI types are shown in Table 2. The distribution of df-ASA for different types is presented in Supplementary Figure One [see Additional file 2]. It is possible to observe a distinct difference in the distribution of non-obligate (i.e., ENZ and nonENZ) and obligate (i.e., HET and HOM) complexes. The distribution patterns of *dom-face *area for ENZ are similar to those of nonENZ and the same trend occurs between HET and HOM. In the distribution of the area of interaction sites, obligate PPI types exhibit a greater variance and in general tend to have larger interaction sites than non-obligate complexes.

**Table 2:** Average values of the properties

Type	ASA(Å^2^)	HH	inPro	nAtom	nAA	nSSE	LCS	nFrag
ENZ	860.42	0.40	0.596	121.73	33.71	11.22	3.3	12.32
nonENZ	823.06	0.37	0.530	106.89	29.59	12.91	2.5	12.91
HET	2237.92	0.41	0.982	344.26	82.56	21.35	3.5	21.35
HOM	1306.37	0.42	0.262	184.55	48.14	13.00	2.9	16.78

The average hydrophobicity (HH) values for ENZ, nonENZ, HET and HOM are respectively 0.40, 0.37, 0.41, and 0.42. Even though average HH values are similar for different PPI types, the histogram distributions of hydrophobicity (see Supplementary Figure Two [see Additional file 2]) exhibit distinctive separation patterns between non-obligate and obligate interactions. The distribution of HH for ENZ is similar to nonENZ and that of HET is similar to HOM.

We note that Arg, His, Tyr, Gln and Trp exhibit higher propensities than other amino acids, while Gly has a low propensity in our analysis. Average residue propensities are shown in Table 2. HET has the highest residue propensity and HOM the lowest. We also analyzed the top four frequent residues for each interaction type calculating the sum of ASA for each amino acid (results in Supplementary Figure Three [see Additional file 2]). Hydrophobic residues including Leu, Ala, and Val frequently occur in types HET and HOM. The charged residue Glu also appears frequently in HET. In nonENZ, charged residues including Asp, Glu, Lys, and Arg are present in the top four frequent residues.

ENZ includes not only some polar residues Ser and Tyr but also the charged residue Glu. We observed that the charged residues occur very frequently in all interaction types and appear dominantly in HET. Trp, Cys, and Met rarely occurred in interface area through all types.

The average values of the size ratio between *dom-face *area and domain area for ENZ, nonENZ, HET, and HOM are 27.03, 20.67, 31.94, and 23.26 respectively as shown in Table 2. The distribution of size ratio is shown in Supplementary Figure Four [see Additional file 2]. We observed that 92% of *dom-faces *are smaller than a half of their domain sizes based on the calculation of ASA values. The average length of consecutive residues (LCS) are 3.3, 2.5, 3.5 and 2.9 for ENZ, nonENZ, HET, and HOM respectively as shown in Table 2.

**Figure 1:**
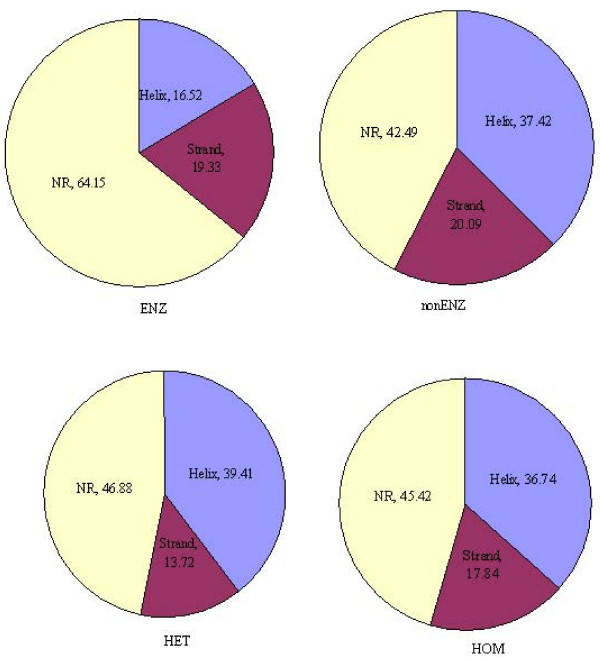
**Distribution of SSE content**. The average distribution of SSE content is distinctive among different PPI types. More than 40% of atoms in interaction sites for all PPI types are positioned in non-regular regions. Interaction sites contain higher portion of non-regular regions than those of helix and strand regions. Especially, less than 20% of interaction sites are composed of strands.

The average distribution of SSE elements (helix, strand and non-regular regions) for different PPI types is shown in Figure 1. We have seen that interaction sites are mostly composed of non-regular regions followed by helix and strand regions. ENZ contains 64.15% of non-regular regions, which is the highest percentage. Helix content are greater than 36% in types nonENZ, HET and HOM but are less than 17% in ENZ. Strand content for all types are less than 20% and HET exhibits the lowest value (13.72%).

The variation in the number of amino acids (nAA) is similar to that for the number of atoms (nAtom). Average values for nAtom, nAA, nSSE and nFrag are shown in Table 2. We found that values for Types HET and HOM are higher than for Types ENZ and nonENZ in all these categories. The distribution of these properties (results shown in Supplementary Figure Seven [see Additional file 2]) for ENZ is similar to nonENZ.

### Classification of PPI types

We were able to discover a total of 1,168 rules describing associations by employing ARM. After the pruning stage a total of 157 association rules [see Additional file 3] were selected for the classification process. The number of rules associated with types ENZ, nonENZ, HET and HOM are 65, 49, 19, and 24 respectively (Table 3). A total of 58 of these are unique, i.e. rules exclusively associated with just one PPI type. The remaining 99 rules are overlapping (non-unique) rules related to two or more PPI types. We are interested in this distinction because unique rules appear to be related to specific characteristics of PPI types, whilst overlapping rules can be related to common attributes of different interaction types or for instance to distinctive properties between obligate and non-obligate interactions.

**Table 3:** The number of association rules discovered for each PPI type

Type	#. of Domains^*a*^	#. of Rules^*b*^	Unique Rules^*c*^	Overlapping Rules^*d*^
ENZ	49	65	34 (52.31%)	31 (47.69%)
nonENZ	47	49	16 (32.65%)	33 (67.35%)
HET	33	19	7 (36.84%)	12 (63.16%)
HOM	225	24	1 (4.17%)	23 (95.83%)

Total	354	157	58 (36.94%)	99 (63.06%)

^*a*^#. of Domains: A number of domains in each PPI type;

^*b*^#. of Rules: A number of association rules discovered for each PPI type;

^*c*^Unique Rules: A number of association rules associated with just one PPI type;

^*d*^Overlapping Rules: A number of rules of which bodies are identical to those of rules in other types.

The performance for different classification methods measured as total accuracy over 10 fold cross validation for ARBC is shown in Table 4. Additionally we performed classification based on the physicochemical properties of the different *dom-faces*(CWAR), and also ARBC classification based on a rule profile generated using only the set of 58 unique rules discovered (UR). Performance results for these approaches are also given in Table 4. We have seen that in all these cases SVM exhibited the best performance among diverse classifiers studied, reaching over 99% accuracy in some cases. However this high accuracy suggests that overfitting problems are associated with the use of SVM. The other classification approaches evaluated still exhibit a high accuracy with the exception of NB. The performance reached by them is comparable to that previously reported in [12] although not exactly the same instances and features were employed. Additionally we observed that there was no significant appreciable difference between the performance of ARBC and CWAR in most of the situations, although it seems that CWAR performed slightly better than ARBC.

**Table 4:** Accuracy for difference classification methods

*Method*^*a*^	DT	RF	KNN	SVM	NB
*All data*^1^:					
*ARBC*^*b*^	0.924	0.968	0.943	0.999	0.476
*CW AR*^*c*^	0.926	0.971	0.978	0.999	0.531
*UR*^*d*^	0.873	0.933	0.893	0.970	0.519
					
*No SSE data*^2^:					
*ARBC_WO_SSE*^*e*^	0.917	0.951	0.936	0.992	0.451
*CW AR_WO_SSE*^*f*^	0.927	0.970	0.979	0.988	0.492
*UR_WO_SSE*^*g*^	0.800	0.850	0.800	0.890	0.483

^*a*^*Method *represents different classification methods such as Decision Tree (DT), Random Forest (RF), K Nearest Neighbor(KNN), Support Vector Machine (SVM) and Naive Bayes (NB);

^*b*^*ARBC*: Association rule based classification;

^*c*^*CW AR*: Classification based on physicochemical properties;

^*d*^*UR*: ARBC classification using 58 unique association rules;

^*e*, *f*, *g*^: Data sets with exclusion of SSE content from All *data*^1^;

^1^All data: Data sets including SSE content;

^2^No SSE data: Data sets without inclusion of SSE content.

These results strongly suggest that ARBC performs competitively with conventional classification approaches for this task, and consequently the use of ARBC does not involve an important loss of information derived from ARM. The performance of ARBC using only unique rules clearly decreased for all classification methods evaluated, although maintaining an acceptable accuracy of near or over 90% in most of the cases. This suggest that unique rules can be influential in classifying most of the PPI types considered in our study and that overlapping rules are important to improve the accuracy of the classification task. It is important to emphasize that the aim of our research is focused on the advantage of interpretability of the discovered rules rather than the optimization of the classification task.

We further investigated the influence of SSE information on the classification of PPI types. We evaluated three different data sets without using the secondary structure elements of proteins, including ARBC_WO_SSE, CWAR_WO_SSE and UR_WO_SSE. Each of the two rule profiles in this case contains a total of only 135 association rules and 43 unique rules. Results for these evaluations are also highlighted in Table 4. It was found that in all cases the performance of diverse classifiers tended to decrease when SSE data was omitted, although only a slightly reduction is observed in most of the classifiers evaluated. Interestingly the major decrement in performance was observed when employing UR_WO_SSE, with accuracies of less than 90% for all classifiers including SVM. These results strongly suggest that SSE content in interaction sites could have an important role in the discrimination of different PPI types for both approaches including ARBC and CWAR.

This implies that the average confidences of the rule sets that include this SSE content information may be higher than those without it. There were 14.01% (22 out of 157) such rules that included SSE content information and their average confidence was 0.533 (Table 5). When we considered the top 31 rules that are covered by 20% of all the rules, their confidence was 0.642. Among them, 42% (13 out of 31) contained SSE information with an average confidence of 0.661. The SSE content rules were enriched among those rules exhibiting higher confidences. The same trend was also seen with unique rules: while the average confidence of 58 unique rules was 0.536, that of the 16 unique SSE rules was 0.622. Here we infer that SSE content in interaction sites is a significant feature that permits reliable classification of the interaction types.

**Table 5:** Analysis of SSE content rules over different subsets

Subset	#. of rules	Fraction(%)	$conf_{1}^{a}$	#. of SSE rules	$conf_{2}^{b}$
*SSE*^*c*^	22	14.01%	0.533	-	-
*TOPK*^*d*^	31	19.75%	0.642	13	0.661
*Unique*^*e*^	58	36.94%	0.536	16	0.622

^*a*^*con f*_1_: Average confidence of a rule subset;

^*b*^*con f*_2_: Average confidence of SSE content rules in a rule subset;

^*c*^*SSE*: Association rules encoding SSE content;

^*d*^*TOPK*: Top K rules covering top 20% in confidence;

^*e*^*Unique*: Unique rules.

### Interpretation of Discovered Association Rules

#### Determination of Important Rules

To select a set of informative and discriminative rules for the extraction of knowledge, most of the existing approaches rank the association rules based on the confidence value of a individual rule. A strong rule which is highly confident and represents general knowledge, may not be a good discriminative rule for the classification. Instead, a better measure of the importance of a rule should include the following factors considered together: correlation between a property and a class, the degree of classification power, confidence and support, top K coverage and uniqueness of a rule. As noted in the previous section, the inclusion of the SSE content information in our ARBC approach has a positive effect on the classification accuracy (Table 4). The importance of a rule can be quantified by integrating the various factors including the SSE content information. We defined a importance factor (I in Tables 6 and 7) by an average value of all the factors. In order to illustrate the informativeness of the rules in understanding interface features, some representative rules within the top 30% (ranked higher than 48) of I are listed in Table 6. The list was complemented by some other rules ranked below 48 in order to explain overlapping rules and compare association rules to rules generated from a decision tree. Similarly, rules describing the ENZ type with varying different structural features are listed in Table 7. Rules in Tables 6 and 7 are sorted by Type and I.

**Table 6:** Representative examples of association rules for each type

#^*a*^	O^*b*^	Rule description^*c*^	Type^*d*^	Conf^*e*^	Supp^*f*^	C^*g*^	G^*h*^	K^*i*^	U^*j*^	S^*k*^	I^*l*^
1	3	If 77.31 ≤ Loop < 80.56	ENZ	0.811	0.032	1	0.214	1	1	1	0.722
2	8	If 17.57 ≤ Helix < 20.87	ENZ	0.545	0.032	1	0.102	1	1	1	0.668
3	9	If SCOPClass = 7	ENZ	0.725	0.053	1	0.184	1	1	-	0.660
4	26	If 67.59 ≤ Loop < 70.83	ENZ	0.526	0.032	-	0.048	1	1	1	0.601
5	28	If 461.83 ≤ df-ASA < 681.42 AND 2.3 ≤ LCS < 2.73	ENZ	0.625	0.032	-	0.120	1	1	-	0.555
6	37	If 57.87 ≤ Loop < 61.11	ENZ	0.467	0.037	-	0.045	-	1	1	0.510

7	2	If SCOPClass = 1 AND 12.25 ≤ nFrag < 16 AND NoStrand	nonENZ	0.882	0.032	1	0.250	1	1	1	0.738
8	11	If .66 ≤ inPro < .87	nonENZ	0.597	0.042	1	0.129	1	1	-	0.628
9	15	If 26.74 ≤ nAA < 35.32 AND 901.01 ≤ df-ASA < 1120.6	nonENZ	0.556	0.032	1	0.133	1	1	-	0.620
10	18	If SCOPClass = 1 AND 1.87 <= LCS < 2.3 9	nonENZ	0.545	0.032	1	0.137	1	1	-	0.619
11	20	If 1.43 ≤ LCS < 1.87	nonENZ	0.556	0.042	1	0.074	1	1	-	0.612
12	21	If NoStrand AND 1.87 ≤ LCS < 2.3	nonENZ	0.515	0.037	-	0.113	1	1	1	0.611
13	36	If 58.11 ≤ ASAPR < 59.52	nonENZ	0.476	0.032	1	0.065	-	1	-	0.515
14	38	If 41.67 ≤ Loop < 44.91	nonENZ	0.423	0.032	-	0.046	-	1	1	0.500
15	40	If SCOPClass = 1 AND NoStrand	nonENZ	0.484	0.064	-	0.074	-		1	0.406
16	46	If 125.14 ≤ nAtom < 165.52 AND 901.01 ≤ df-ASA < 1120.6	nonENZ	0.412	0.037	-	0.050	-	1	-	0.375
17	64	If .42 ≤ HH < .44	nonENZ	0.347	0.037	-	0.009	-	1	-	0.348

18	5	If 7.78 ≤ Strand < 10.27	HET	0.660	0.037	1	0.141	1	1	1	0.691
19	7	If 2.8 ≤ Strand < 5.29	HET	0.565	0.037	1	0.089	1	1	1	0.670
20	12	If 205.9 ≤ nAtom < 246.28	HET	0.574	0.037	1	0.143	1	1	-	0.626
21	25	If 44.91 ≤ Loop < 48.15	HET	0.479	0.037	1	0.110	-	1	1	0.604
22	32	If 3.6 ≤ LCS < 4.03	HET	0.461	0.037	1	0.100	-	1	-	0.520
23	33	If .44 ≤ HH < .46	HET	0.467	0.045	1	0.070	-	1	-	0.516
24	63	If SCOPClass = 1 AND NoStrand	HET	0.282	0.037	-	0.074	-	-	1	0.348

25	31	If SCOPClass = 3 AND 2.3 ≤ LCS < 2.73	HOM	0.470	0.033	1	0.100	-	1	-	0.521
26	98	If 3.17 ≤ LCS < 3.6	HOM	0.337	0.035	-	0.034	-	-	-	0.135
27	133	If 26.74 ≤ nAA < 35.32	HOM	0.237	0.039	-	0.041	-	-	-	0.106

Representative examples of 27 rules within top 30% are listed by sorting Columns Type and I. Rules of which order is below 48 are added for explaining overlapping rules and the comparison to rules produced from a decision tree.

^*a*^#: Rule identifier;

^*b*^O: Order of a rule ranking by importance factor;

^*c*^Rule description: The body of a rule;

^*d*^*Type*: The head of a rule representing a PPI type;

^*e*^*Conf*: Confidence of a rule;

^*f *^*Supp*: Support of a rule;

^*g*^*C*: Rules selected from correlation-based feature subset selection [32];

^*h*^*G*: The worth of a rule by measuring the gain ratio [33]with respect to PPI types;

^*i*^*K*: Top K rules ranked within top 30%;

^*j*^*U*: Unique rules;

^*k*^*S*: SSE content rules;

^*l*^*I*: Importance factor of a rule calculated by an average of all factors such as Conf, Supp, C, G, K, U and S; "-" is replaced with value 0 when the importance factor was calculated.

**Table 7:** Representative examples of ENZ type presenting different structural features

#	O	Rule description	Subtype	Conf	Supp	C	G	K	U	S	I
28	24	If NoHelix	ENZ_A, ENZ_B, ENZ_C	0.508	0.069	-	0.058	1	1	1	0.606
29	1	If SCOPClass = 7 AND NoHelix	ENZ_A, ENZ_B	1.000	0.032	1	0.315	1	1	1	0.764
30	17	If 461.83 ≤ df-ASA < 681.42 AND NoHelix	ENZ_A, ENZ_B	0.593	0.037	-	0.085	1	1	1	0.619
31	39	If 461.83 ≤ df-ASA < 681.42	ENZ_A, ENZ_B	0.477	0.111	1	0.076	-	-	-	0.416
32	16	If NoHelix AND nFrag < 4.75	ENZ_A	0.612	0.032	-	0.076	1	1	1	0.620
33	19	If 4.75 ≤ nSSE < 6.62 AND NoHelix	ENZ_A	0.588	0.032	-	0.072	1	1	1	0.538
34	51	If 461.83 ≤ df-ASA < 681.42 AND 4.75 ≤ nSSE < 6.62	ENZ_A	0.417	0.032	-	0.018	-	1	-	0.367
35	77	If 44.38 ≤ nAtom < 84.76 AND 461.83 ≤ df-ASA < 681.42	ENZ_A	0.396	0.058	-	0.023	-	-	-	0.159
36	34	If 9.58 ≤ nAA < 18.16 AND 44.38 ≤ nAtom < 84.76 AND 461.83 ≤ df-ASA < 681.42	ENZ_A	0.500	0.032	-	0.045	1	1	-	0.515
											
37	60	If 18.16 ≤ nAA < 26.74 AND 44.38 ≤ nAtom < 84.76	ENZ_A	0.357	0.032	-	0.015	-	1	-	0.351
38	10	If 84.76 ≤ nAtom < 125.14 AND 461.83 ≤ df-ASA <681.42	ENZ_B	0.617	0.053	1	0.145	1	1	-	0.636
39	13	If 12.66 ≤ sRatio < 15.06 AND 461.83 ≤ df-ASA < 681.42	ENZ_B	0.600	0.032	1	0.113	1	1	-	0.624
40	14	If 461.83 ≤ df-ASA < 681.42 AND 10.38 ≤ nSSE < 12.25 AND SCOPClass = 2	ENZ_B	0.857	0.032	-	0.230	1	1	-	0.624
41	27	If SCOPClass = 2 AND 461.83 ≤ df-ASA < 681.42 AND 84.76 ≤ nAtom < 125.14	ENZ_B	0.789	0.032	-	0.176	1	1	-	0.599
											
42	35	If 10.38 ≤ nSSE < 12.25 AND 12.25 ≤ nFrag < 16	ENZ_B	0.500	0.032	-	0.043	1	1	-	0.515
43	73	If 84.76 ≤ nAtom < 125.14 AND SCOPClass = 2	ENZ_B	0.408	0.042	-	0.043	-	-	-	0.164
44	114	If 84.76 ≤ nAtom < 125.14 AND 26.74 ≤ nAA < 35.32	ENZ_B	0.307	0.037	-	0.024	-	-	-	0.123
45	109	If 681.42 ≤ df-ASA < 901.01	ENZ_C	0.317	0.048	-	0.013	-	-	-	0.126
46	137	If 84.76 ≤ nAtom < 125.14 AND 681.42 ≤ df-ASA < 901.01	ENZ_C	0.252	0.032	-	0.009	-	-	-	0.098
											
47	146	If SCOPClass = 4	ENZ_C	0.221	0.042	-	0.011	-	-	-	0.091
48	101	If 35.32 901.01 nAA < 43.9 AND 125.14 ≤ nAtom < 165.52	ENZ_D	0.323	0.032	-	0.041	-	-	-	0.132
49	130	If SCOPClass = 3	ENZ_D	0.238	0.069	-	0.016	-	-	-	0.108
50	141	If 901.01 ≤ df-ASA < 1120.6	ENZ_D	0.207	0.032	-	0.050	-	-	-	0.096
51	54	If 1120.6 ≤ df-ASA < 1340.19	ENZ_E	0.392	0.042	-	0.018	-	1	-	0.363

Abbreviation of column names is the same as that of Table 6.

The ENZ subtypes are defined in Figure 4. Note that ENZ_B includes both inhibitors and enzymes while the others are exclusively formed by inhibitors (e.g. ENZ_A, ENZ_C and ENZ_E) or enzymes (e.g. ENZ_D).

**Figure 2:**
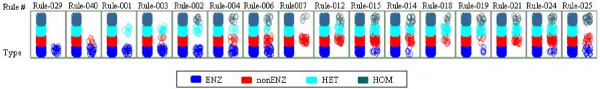
**A scatter Plot matrix for PPI types and association rules**. This scatter plot matrix shows clusters as collection of points separated by association rules encoding SSE content information or a SCOP class. Different colors of the left in each plot (a cell) correspond to four PPI types. The right of a plot area presents the distribution of points met with a rule on the head of a cell. Rules 29, 40, 1, and 3 separate ENZ and nonENZ from other types remarkably with few errors. The Rule 29 is a strong discriminator to classify ENZ from other types completely.

**Figure 3:**
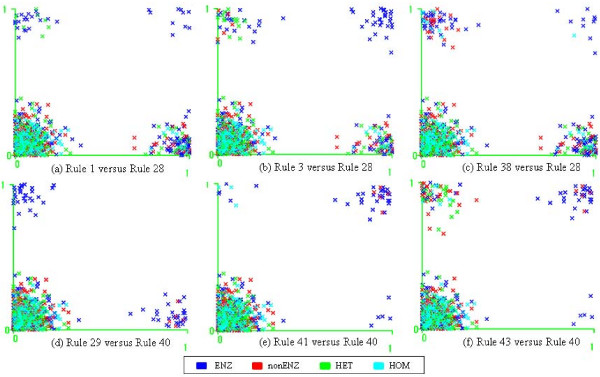
**2D plots for pairs of association rules**. These plot data points by pairs of association rules. X and Y axes are a pair of rules and each of them have two boolean values. 0 represents negative data points not meeting with a rule of each axis and 1 represents for positive data points meeting with the rule. The data points on the upper left corner meet a rule used for Y axis and the data points on the down right corner meet a rule used for X axis. The points on the upper right corner meet with both rules used for X and Y axes. Plots in Figure 3(a), (b), and (c) characterize distribution of inhibitors in enzyme-inhibitors interactions. Rule 28 is used for X axis in plots (a), (b) and (c). Rules 1, 3 and 38 are used for the Y axis in those plots. (a) represents an example for a pair of rules both including SSE information (e.g. helix and loop content). (b) and (c) show examples for combination of SSE content information (Rule 28: "*Nohelix *") with other properties (e.g. SCOPClass, number of atoms and etc.). Plot (b) (Rule 3 versus Rule 28) is identical to the plot generated by Rule 29. Enzymes interacting with a group of inhibitors characterized by (a), (b), and (c) are featured by in Figure 3(e), and (f). Enzymes and inhibitors described by Rules 40 and 29 respectively are plotted in (d) where there is no point matching with both rules. Plot (d) reflects proper interpretation of association rules regarding interactions between enzymes and inhibitors.

We have shown that the interaction sites were dominated by non-regular region: especially for ENZ interactions, almost $\frac{2}{3}$ of the sites in average were composed of non-helix and non-beta strand regions (Figure 1). This is manifested in rules 29 (Table 7), 1, 4 and 6, all of which require 50 – 80% content of non-regular regions to be classified as ENZ. Some of the rules containing negation predicates are strong indicators of certain interaction types. For example, "*Nohelix *" and "*Nostrand *" in the interaction sites imply ENZ (Rule 29) and nonENZ (Rules 7, 12 and 15), respectively. HET is characterized by relatively small portions of strands (Rules 18, and 19) and "*Nostrand *" (Rule 24). It is also observed that rules containing such SSE content information conjuncted with other properties (Rules 29, 7, 12, 15 and 24 in Figure 2) or combined with other rules (Figure 3(a), (b) and 3(c)) become stronger discriminators for classifying PPI types than rules containing only SSE content information (Rules 1, 2, 4, 6, 14, 18, 19 and 21 in Figure 2). We note that some rules (Rules 29 and 7 in Figure 2) containing SSE information with SCOP classes are the most discriminative and informative in order to characterize ENZ and nonENZ.

#### Inference of Subtypes

**Figure 4:**
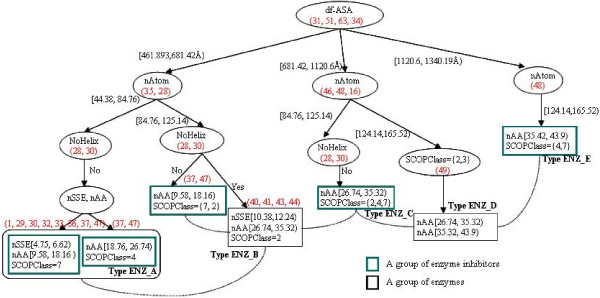
**A hierarchical tree for supporting inference of subtypes**. A hierarchical tree drawn from association rules (Table 7) represents different structural groups in ENZ. Enzyme-inhibitor interactions are characterized with size scales of interaction sites (number of atoms and df-ASA) and SSE content information (helix content). These differences of structural groups result in subtypes of PPIs. Letters in red are identifiers of rules (Tables 6 and 7) to split branches of a tree. Dashed lines show interaction between enzymes and inhibitors in different subtypes.

Some rules which share the same sets of properties but differ in their value ranges or have other properties can be effective in order to compare features of different interaction types or to identify subtypes in a PPI type. For example, among the top 30% rules, Rules 38 (Table 7) and 16 (Table 6) describe types ENZ and nonENZ respectively, using the same set of properties such as number of atoms and df-ASA. However, their values imply that the interaction sites of nonENZ (Rule 16) are larger than those of ENZ (Rule 38). The ranges of size scales of interaction sites in ENZ are presented in Rules 35, 38 and 46 (Table 7) that share the same set of properties but differ in their values. The overall size of interaction sites in ENZ are described by Rule 38 with the highest confidence among those rules encoding the size of interaction sites. These are interesting cases where the structural difference between types can be directly inferred and subtypes of a PPI type can be derived by grouping different features of interaction sites. We deduced five subtypes of ENZ and a hierarchical tree (Figure 4) to account for those subtypes. We compiled a list of representative association rules (Table 7) to show structural features different among these subtypes.

We note that interaction sites of enzymes are distinguished from those of inhibitors in enzyme-inhibitor complexes. Interaction sites for inhibitors are relatively small, i.e., mainly < 1000 *Å^2 *(Rules 34, 35, 37, 38 and 46), and are made up of strands (Rule 41) and mostly non-regular regions (Rules 1, 4 and 6) without helix content (Rule 3, 28, 29, 30, 32, and 33) which is very informative in order to characterize inhibitors. Remarkably Rules 30 and 28 generalize common features of inhibitors with respect to the size of interaction sites and SSE content. As Rule 29 was considered to be very discriminative to differentiate ENZ from other types, it can depict characteristics of a small group of inhibitors with indicating that inhibitors in SCOP class 7 do not contain helix in interaction sites (Figure 3(a), (b) and 3(c)).

In contrast, enzymes have larger interaction sites than their inhibitors and form mixtures of helices and strands in interaction sites (Rules 40, 48, 49, 50 and 51). Both Rules 33 and 40 show that enzymes (Rule 40) have SSEs twice as many as inhibitors (Rule 33). This indicates that both enzymes and inhibitors may contain mainly strands as regular SSEs in interaction sites since enzymes are included in SCOP class 2 (mainly *β*) and inhibitors do not contain helices in interaction sites. This suggests that non regular regions and beta strands are mainly involved in the interfaces of enzyme-inhibitor interactions. Such extracted information can be useful for the prediction of interaction sites for enzyme-inhibitor complexes. This observation is demonstrated by some small inhibitors in Type ENZ_A (1tabi_, 2ptci_, and 4sgbi_) and Type ENZ_B (1*mcti_*). Those inhibitors interact with enzymes in Type *ENZ_B*. The enzymes described by Rules 40, 41 and 43 are included in SCOP superfamily trypsin-like serine proteases (2.47.1) and the inhibitors are mainly in SCOP class 7 which is composed of small proteins dominated by metal ligand, heme, and disulfide bridges.

It is possible in a similar way to infer subtypes of other PPI types. Among PPI types, ENZ has plenty of rules (a total of 65) to derive subtypes. Hence, the comparative analysis of association rules was presented for ENZ.

#### Comparison of Association Rules to PART Rules

To improve our understanding of the association rules discovered, we compared PART rules produced from a decision tree built using C4.5 over our properties with the association rules. There were a total of 44 PART rules generated and their average confidence and support were 0.99 and 0.02 respectively. We have collected a representative list of PART rules in Table 8. In the comparison of the association rules with PART rules, PART rules are more complicated with the composition of more predicates in rule bodies than those in association rules. Typically, one PART rule corresponds to more than 2 ~3 association rules (Table 8). Both rules provided quantitative descriptions. However, property values in PART rules represent split points for classification and are not represented by intervals of quantitative values. Some PART rules (Rules 1, 3 and 38 in Table 8) including identical properties with different split points in the same rule bodies were not clear enough to determine decision boundaries of properties. These limit the readability and understandability of PART rules whilst the association rules were simple enough to be interpreted by users. It was also possible with association rules to support the comparative analysis of rules between different PPI types as we inferred the possibility of subtypes and relative information by comparison of size scales of interaction sites in ENZ. A set of association rules discovered by ARM comprises mostly weak rules together with a small number of strong rules. On the contrary, most PART rules consist of a number of very strong rules which have the highest confidences and low supports.

**Table 8:** PART rules generated by decision trees using *C*4.5^*a*^

#^*b*^	Rules discovered by C4.5 Decision Tree	Type	Conf	Supp	Corresponding rules^*c*^
5	AVGASA > 68.73025 AND nAtom > 60 AND LCS > 2.61 AND Strand ≤ 32.857 AND SCOPClass = 7	ENZ	1	0.03	35, 5, 3, 36
38	sRatio ≤ 29.411765 AND HH > 0.277096 AND SCOPClass = 2 AND Strand > 16.949 AND Strand > 21.324 AND nSSE > 10	ENZ	1	0.02	40, 39
4	Loop > 50.299 AND nAtom > 60 AND Helix ≤ 33.636 AND AVGASA ≤ 41.137133	ENZ	0.99	0.07	35, 6
27	inPro ≤ 2.016077 AND Helix > 48.485 AND LCS > 1.727 AND Strand ≤ 8.571 AND SCOPClass = 1 AND AVGASA ≤ 53.133	nonENZ	1	0.02	8, 10
40	SCOPClass = 1 AND Strand ≤ 2.26	nonENZ	1	0.01	15
1	nAtom > 189 AND Loop ≤ 66.316 AND nSSE > 13 AND Helix ≤ 19.481 AND sRatio ≤ 80.833 AND inPro > -1.570 AND LCS > 3.714 AND Loop ≤ 46.7	HET	1	0.05	20, 21
3	nAtom > 212 AND Strand ≤ 10.738 AND nSSE > 13 AND inPro > -1.476973 AND nAtom > 384	HET	1	0.05	20, 18, 19
34	SCOPClass = 3 AND Helix > 18.421	HOM	1	0.02	25
15	HH > 0.433 AND AVGASA > 55.984 AND nAA ≤ 34	HOM	1	0.01	27

^*a*^: A total of 44 rules produced by a decision tree using C4.5 algorithm in WEKA machine learning library;

^*b*^#: PART rule identifier;

^*c*^Corresponding rules: Association rule identifiers (Tables 6, 7 and 8) corresponding to a PART rule.

One of the most notable differences between association rules and PART rules is in how to handle overlapping rules between different types. If two different interaction types are predicted from the identical head of a rule, these are called overlapping rules. There were 99 such cases out of a total of 157 rules (Table 3). Their distribution is illustrated in Supplementary Figure Nine [see Additional file 2]. Table 9 shows representative examples of overlapping rules. Examination of the overlapping rules shared by ENZ and nonENZ indicated that these types are similar in terms of df-ASA, nAtom, and nAA (Table 9) differentiated by combination with the rest of properties such as SSE content, average length of consecutive residues, size ratio, and hydrophobicity. PART rules are unique cross PPI types.

**Table 9:** Representative examples of overlapping association rules

#^*a*^	#^*b*^	Rule description^*c*^	Types^*d*^	Conf^*e*^	Supp^*f*^	Conf^*g*^	Supp^*h*^
52	43	If 84.76 ≤ nAtom < 125.14 AND SCOPClass = 2	ENZ^1 ^OR nonENZ^2^	0.408	0.042	0.306	0.032
53	35	If 44.38 ≤ nAtom < 84.76 AND 461.83 ≤ df-ASA < 681.42	ENZ^1 ^OR nonENZ^2^	0.396	0.058	0.252	0.037
54	48	If 35.32 ≤ nAA < 43.9 AND 125.14 ≤ nAtom < 165.52	ENZ^1 ^OR nonENZ^2^	0.323	0.032	0.376	0.037
55	46	If 84.76 ≤ nAtom < 125.14 AND 681.42 ≤ df-ASA < 901.01	ENZ^1 ^OR nonENZ^2^	0.252	0.032	0.336	0.042
56	26	If 3.17 ≤ LCS < 3.6	HET^1 ^OR HOM^2^	0.357	0.037	0.337	0.035

Examples of overlapping rule are selected from Tables 6 and 7.

^*a*^# Rule identifier;

^*b*^#: Rule identifier in Tables 6 and 7;

Rule description^*c*^: The body of overlapping rules between the two types;

^*d*^*Types*: PPI *Type*^1 ^and *Type*^2 ^having overlapping rules in common;

^*e*, *g*^*Con f*: Confidences of overlapping rules for *Type*^1 ^and *Type*^2 ^respectively;

^*f*, *h*^*Supp*: Supports of overlapping rules for *Type*^1 ^and *Type*^2 ^respectively.

## Conclusion

We have developed a classification method that categorizes each PPI into one of four different types using association rule based classification (ARBC). The application of association rule mining over 354 known PPI domains using 14 properties yielded a total of 157 rules, which in turn discriminated the features of interaction sites for different PPI types and were used to generate a classification model to predict PPI types. Our ARBC approach performed competitively compared with conventional methods applied directly to the property values: for example, the work in [12] reported an accuracy of 91.8% for the classification of three types of interactions by directly applying SVM. Although it is not possible to make a direct comparison of their method with ours due to heterogeneity of the data set, this suggests that the processes of association rule generation and subsequent pruning do not incur a loss of relevant information. At the same time, our results demonstrated that we were able to considerably improve the accuracy of the prediction of PPI types through the use of structural domain information for the description of interaction interfaces, and also the use of secondary structure content. Although SSE content alone could not classify interaction sites with high accuracy, its incorporation with other properties improved the accuracy of classification.

Our approach based on ARBC has a clear advantage over conventional methods because results are reported in terms of rules that are a quantitative description of properties and hence their interpretation is straightforward and simple. Thus, biologists can easily judge if a discovered rule is interesting or not. Analysis of common and unique properties together is a unique feature of our approach, unlike conventional classification methods which typically capture unique properties only. Common rules capture those properties which are common between PPI types. In particular enzyme inhibitor (ENZ) and non-enzyme inhibitor (nonENZ) interactions, both being non-obligate or transient, share more properties in common than with other types. As we have demonstrated, all these features produce descriptive rules, enabling their simple and powerful interpretation. We observed that the property distributions of homo-obligate interactions are similar to those of hetero-obligate interactions but distinct from those of non-obligate interactions. We found that obligate interactions have larger and more hydrophobic interaction sites than non-obligate interactions. Hydrophobic residues including Leu, Ala, and Val were found more frequently in obligate interactions whilst polar residues including Ser and Gly were present in non-obligate interactions. Charged residues (Glu, Asp, Lys, and Arg) were seen frequently in all interaction types. On the basis of a detailed analysis of association rules, it was observed that interactions between enzymes and their inhibitors were separated into several different structural subgroups. This may lead to the possibility of different subtypes of PPIs being involved in transient interactions. Our findings based on the interpretation of association rules are consistent with the description of obligate complexes in previous studies [17,12].

In future work we plan to improve our approach by incorporating additional properties such as energy functions and electric potentials for the generation of more accurate and meaningful association rules. The unique contribution of our work is the development of a novel methodology that analyzes specificities and commonalities for interaction types, and we intend to extend this to the prediction of interaction partner and interaction sites.

## Authors' contributions

SHP developed the concept and the method under the supervision of DRG. JAR carried out classification and participated in drafting the paper. SK interpreted the results for the point of view of a biologist. JWK developed the web application. SHP drafted the paper, JAR, DRG and SK finalized the draft. All authors read and approved the final manuscript.

## Supplementary Material

Additional file 1 **Calculation of residue propensity.** A table shows AA propensity for 20 amino acids and a equation represents the calculation of residue propensity. Click here for file

Additional file 2 **Association rules. **A set of association rules discovered for all types presents and rules are sorted by Type and I. Click here for file

Additional file 3 **Distribution of dom-face properties.** The figures represent the statistical distributions of dom-face properties for four PPI types. Click here for file
