# Supplementary material for: Prediction of protein-protein interaction types using association rule based classification
Source: BMC Bioinformatics. 2009 Jan 28;10:36. doi: 10.1186/1471-2105-10-36 (PMC2667511; doi:10.1186/1471-2105-10-36)
Supplement: Additional file 1 — Calculation of residue propensity. A table shows AA propensity for 20 amino acids and a equation represents the calculation of residue propensity. [file 1471-2105-10-36-S1.pdf]

EQUATION

Equation 1

$$AAPropensity = \frac{\sum_{i=1}^{Ni} ASA(AA_k(i)) / \sum_{i=1}^{Ni} ASA(AA(i))}{\sum_{s=1}^{N} ASA(AA_k(s)) / \sum_{s=1}^{N} ASA(AA(s))} \qquad (1)$$

$\sum_{i=1}^{Ni} ASA(AA_k(i))$: sum of the SASA in the monomer of AA type k in the *dom-face*
$\sum_{i=1}^{Ni} ASA(AA(i))$: sum of the SASA in the monomer of all AAs of all types in the interface
$\sum_{s=1}^{N} ASA(AA_k(s))$: sum of the SASA in the monomer of AA of type k on the surface (exterior plus interface residues)
$\sum_{s=1}^{N} ASA(AA(s))$: sum of the SASA in the monomer of all AAs of all types on the surface

TABLE

Table 1. Hydrophobicity scale and Amino Acid Propensity

AA	Hydrophobicity(HI_{AA})	AA Propensity
ARG	0.000	0.289
HIS	0.350	0.254
TYR	0.604	0.225
GLN	0.242	0.154
TRP	1.000	0.137
MET	0.687	0.127
PRO	0.531	0.093
PHE	0.859	0.084
GLU	0.113	0.055
THR	0.390	0.021
ASP	0.074	0.021
LYS	0.006	0.013
ASN	0.126	-0.008
SER	0.298	-0.034
CYS	0.782	-0.083
LEU	0.831	-0.107
ILE	0.862	-0.127
VAL	0.684	-0.127
ALA	0.405	-0.184
GLY	0.310	-0.200
