# Supplementary material for: Prediction of protein-protein interaction types using association rule based classification
Source: BMC Bioinformatics. 2009 Jan 28;10:36. doi: 10.1186/1471-2105-10-36 (PMC2667511; doi:10.1186/1471-2105-10-36)
Supplement: Additional file 2 — Association rules. A set of association rules discovered for all types presents and rules are sorted by Type and I. [file 1471-2105-10-36-S2.pdf]

FIGURES

Type1, Type2, Type3 and Type4 referred to ENZ, nonENZ, HET and HOM respectively.

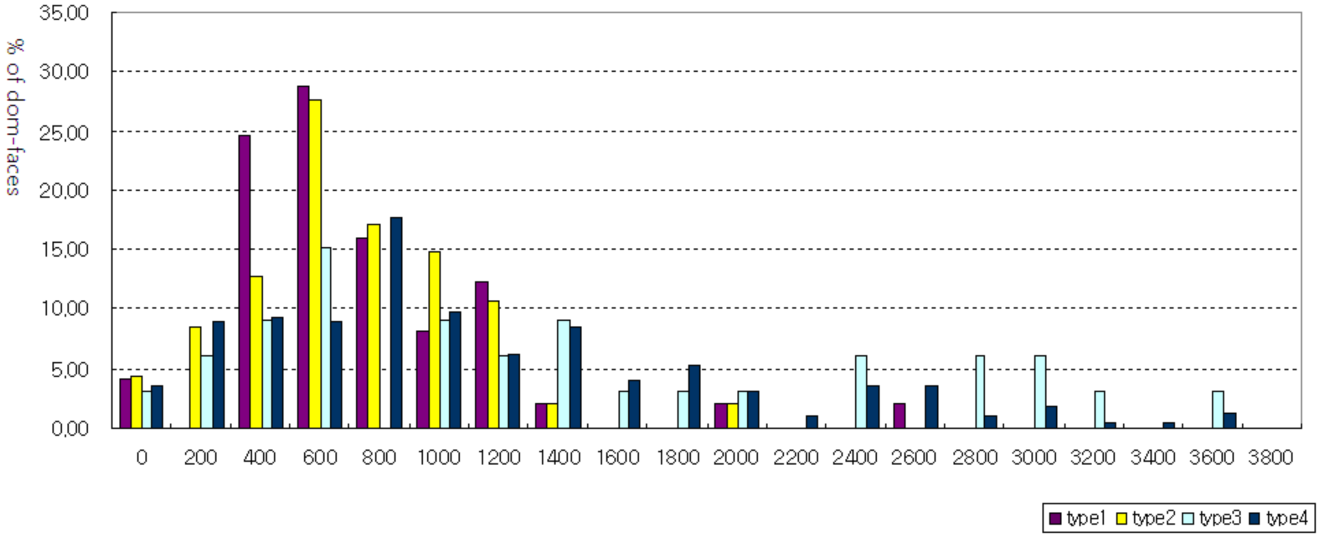

Fig. 1. ASA for *dom-face* area

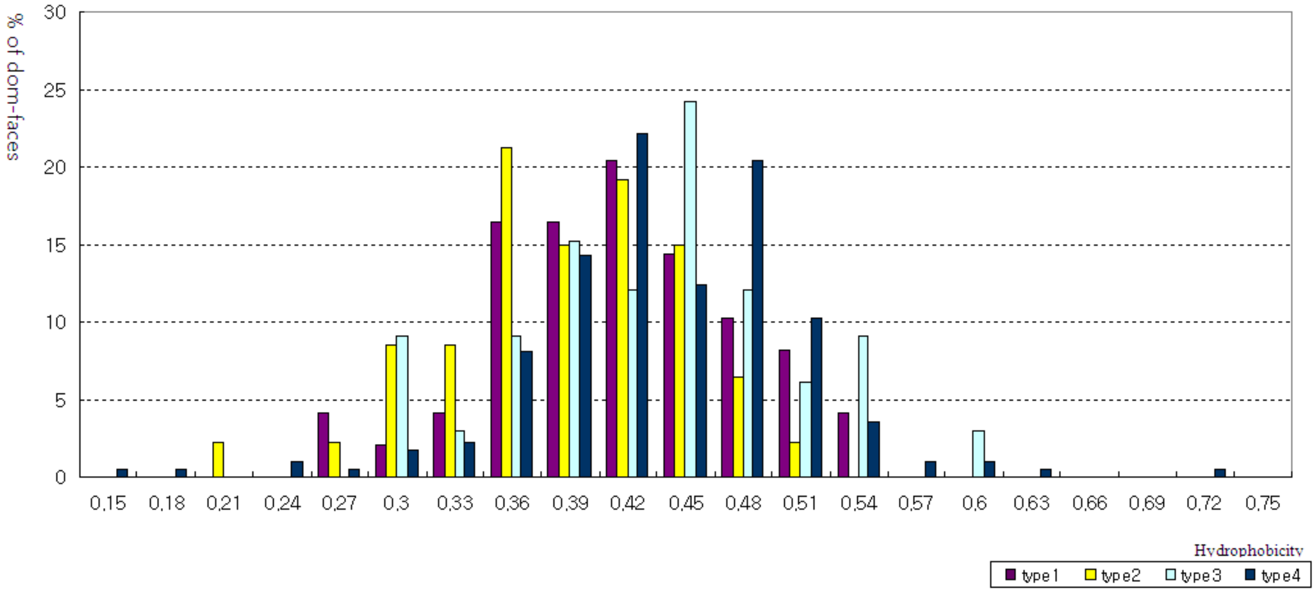

Fig. 2. Hydrophobicity

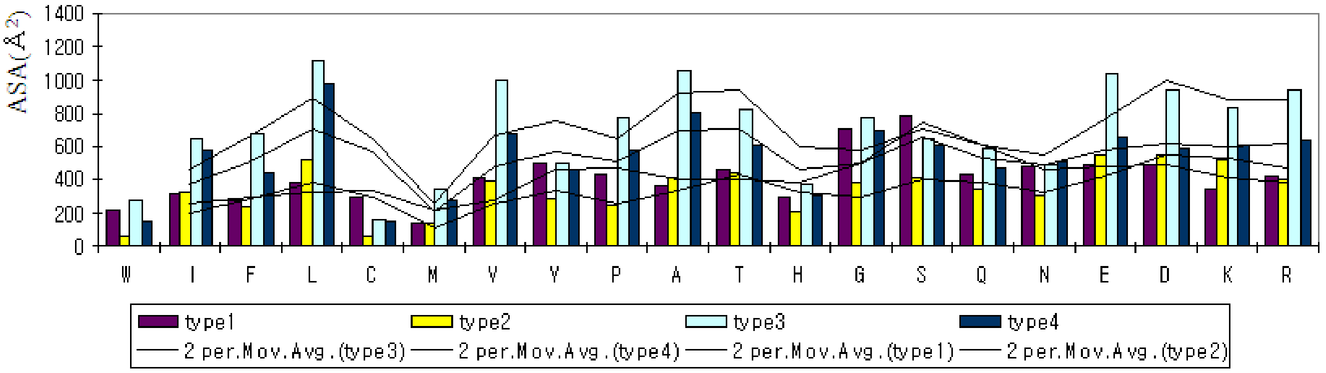

Fig. 3. Amino Acid composition

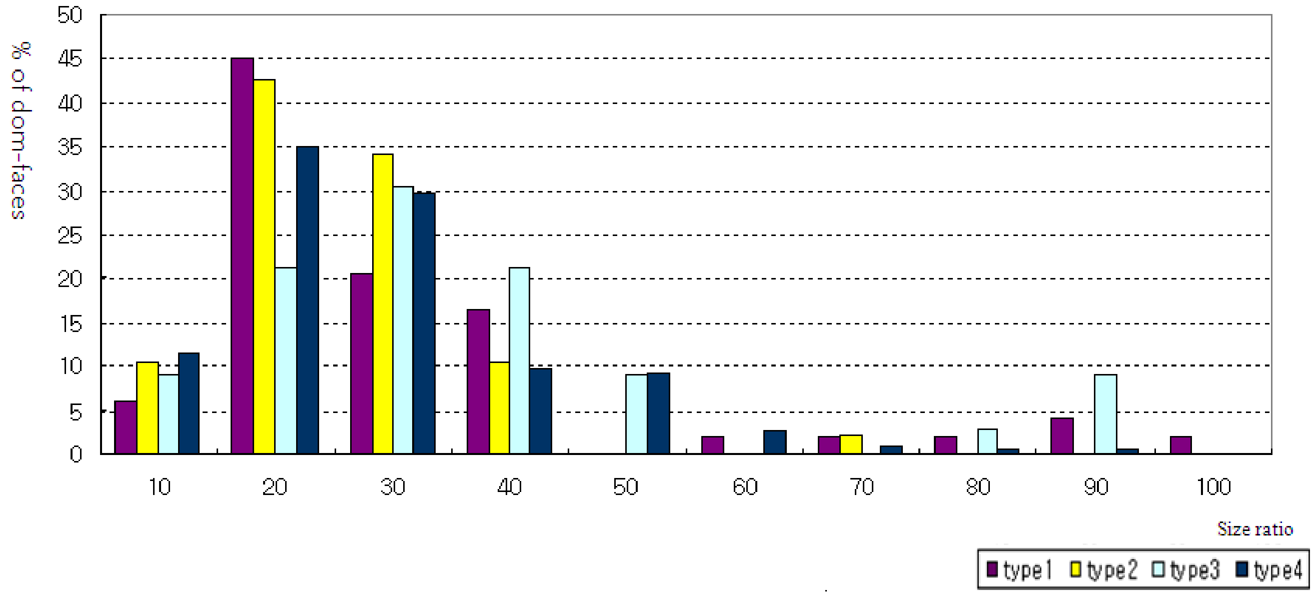

Fig. 4. Distribution of size ratio

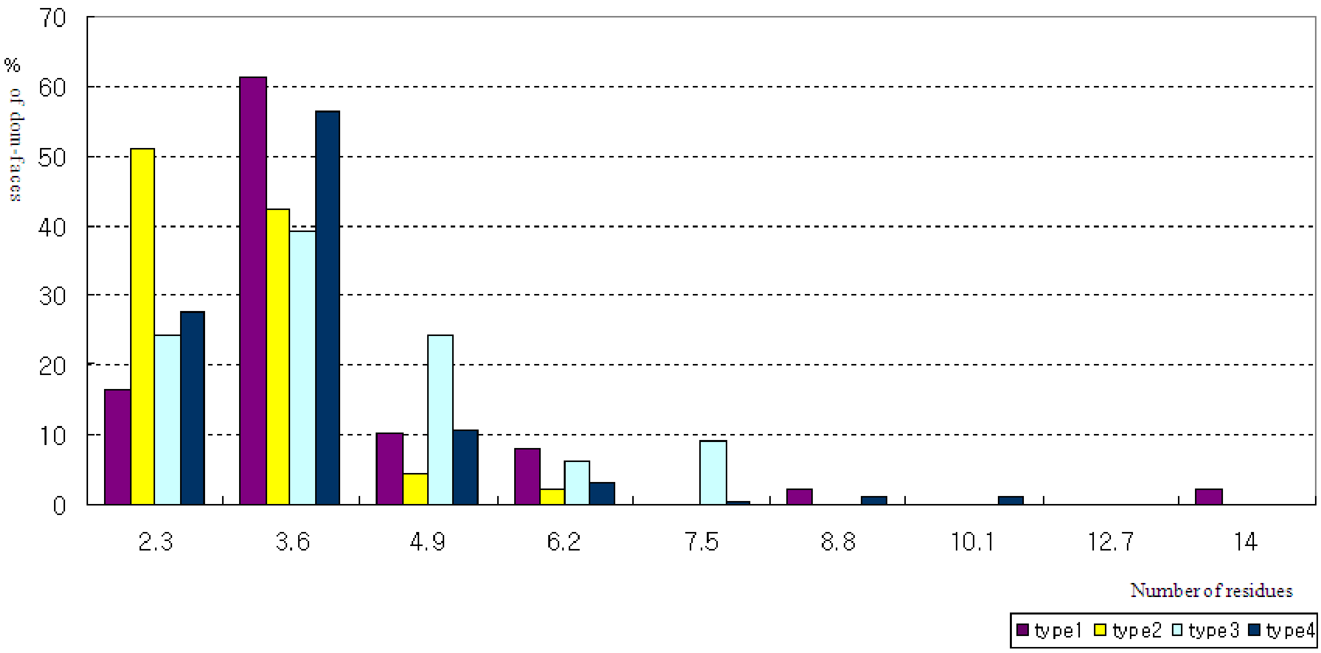

Fig. 5. Distribution of LCS

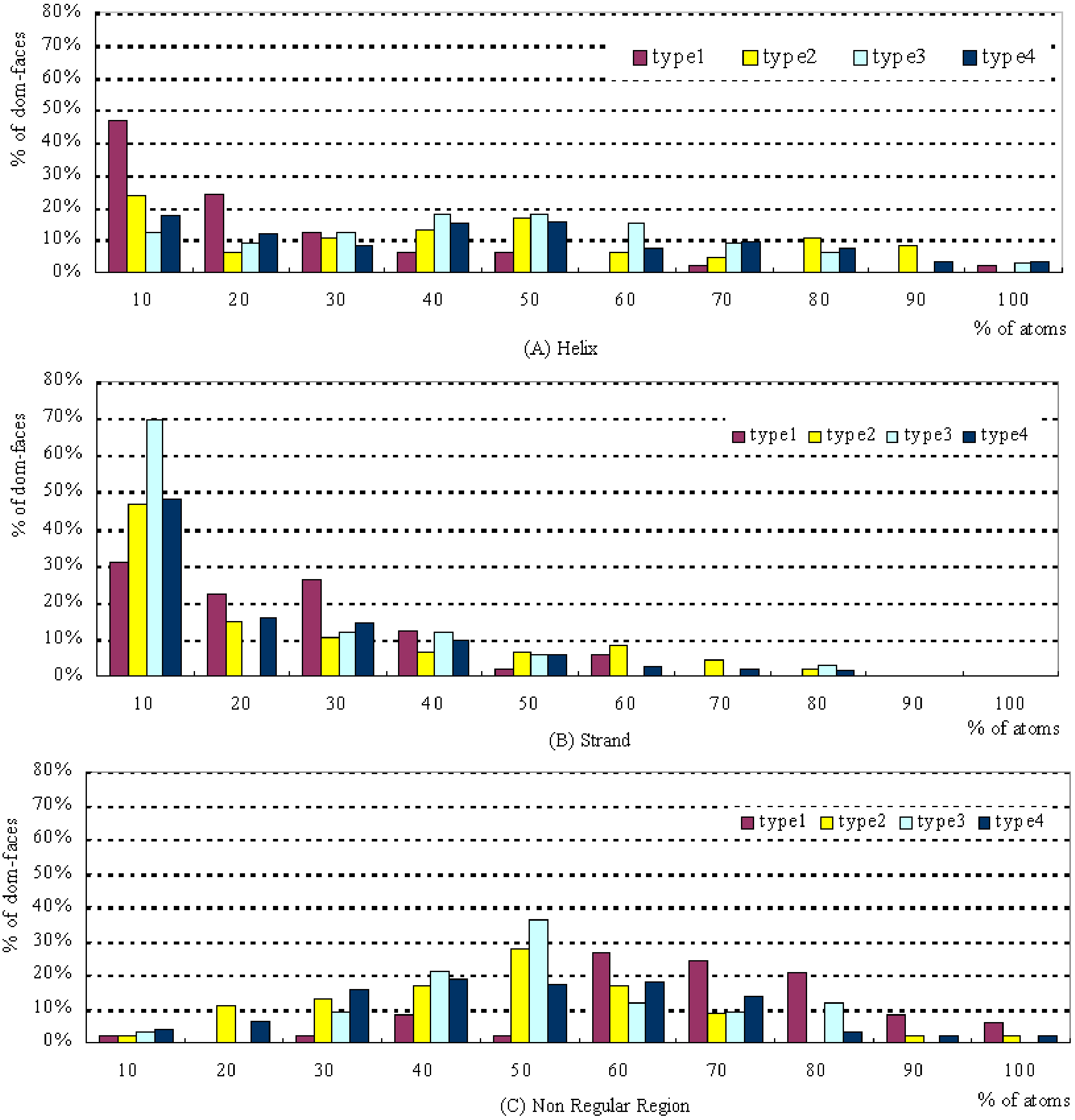

Fig. 6. Distribution of SSE contents

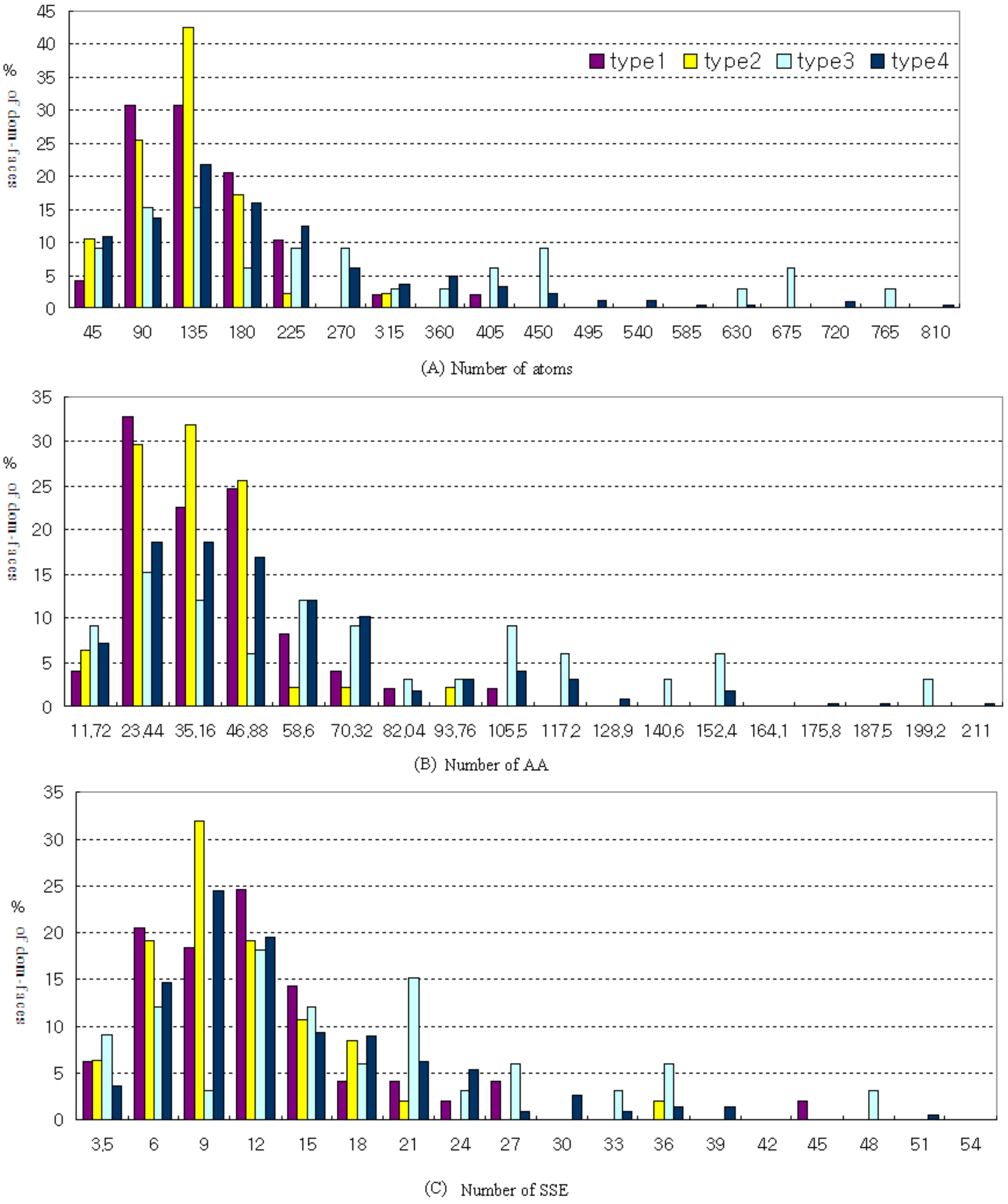

Fig. 7. Distribution of nAtoms, nAAs, nSSEs

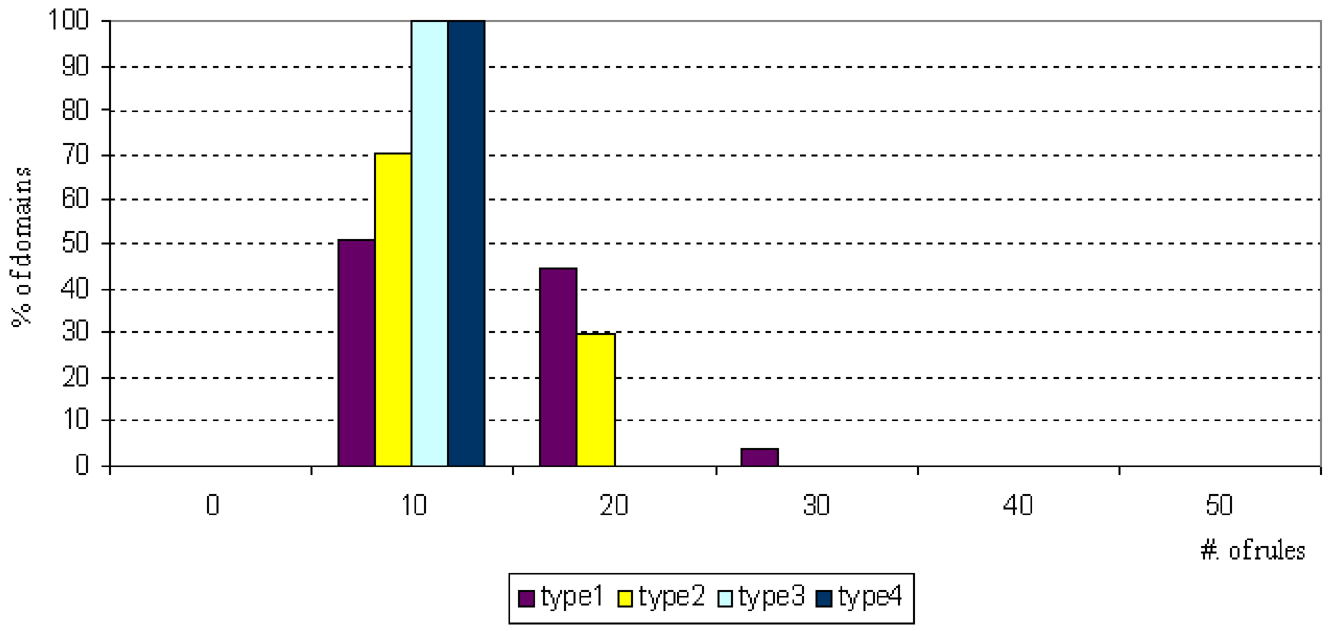

Fig. 8. Distribution of number of domains encoded in a rules

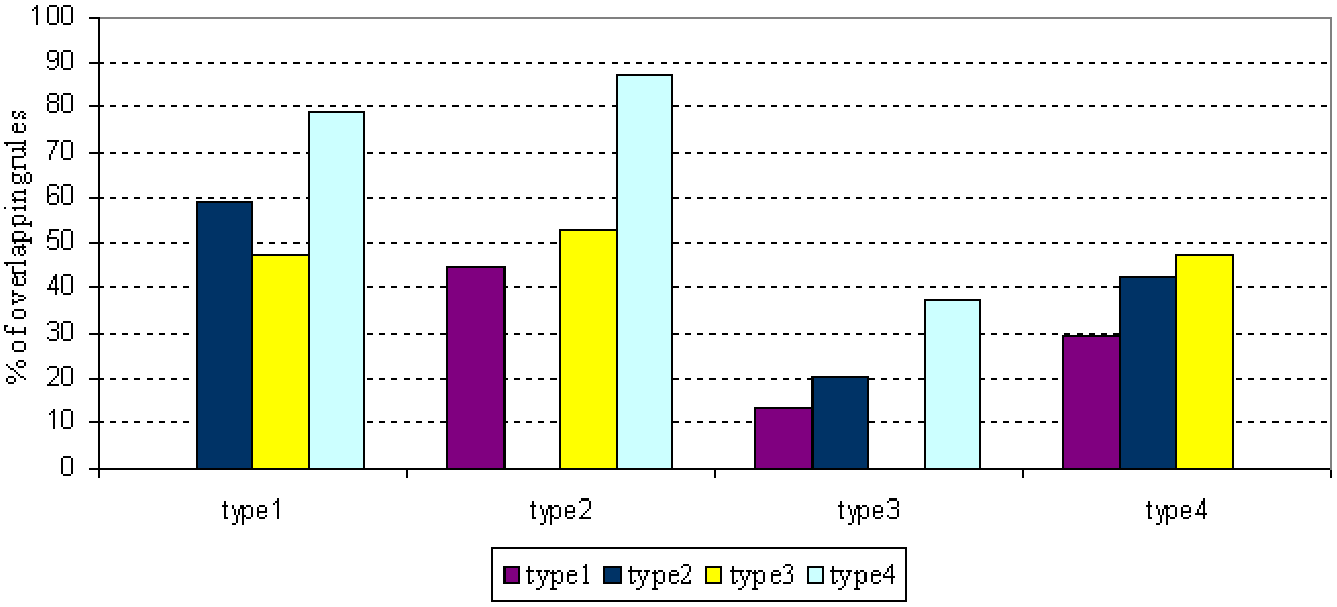

Fig. 9. Distribution of overlapping rules across interaction types
