# Supplementary material for: Prediction of protein-protein interaction types using association rule based classification
Source: BMC Bioinformatics. 2009 Jan 28;10:36. doi: 10.1186/1471-2105-10-36 (PMC2667511; doi:10.1186/1471-2105-10-36)
Supplement: Additional file 3 — Distribution of dom-face properties. The figures represent the statistical distributions of dom-face properties for four PPI types. [file 1471-2105-10-36-S3.pdf]

Association Rules

A list of 157 association rules

O	Rule description	type	conf	supp	C	G	T	U	S	I
1	If SCOPClass = 7 AND NoHelix	ENZ	1	0.03	1	0.315	1	1	1	0.764
2	If SCOPClass = 1 AND 12.25 <= nFrag < 16 AND NoStrand	nonENZ	0.88	0.03	1	0.25	1	1	1	0.738
3	If 77.31 <= Loop < 80.56	ENZ	0.81	0.03	1	0.214	1	1	1	0.722
4	If 12.25 <= nFrag < 16 AND NoStrand	nonENZ	0.79	0.03	1	0.22	1	1	1	0.720
5	If 7.78 <= Strand < 10.27	HET	0.66	0.04	1	0.141	1	1	1	0.691
6	If 461.83 <= df-ASA < 681.42 AND 10.38 <= nSSE < 12.25	ENZ	0.86	0.03	1	0.23	1	1		0.686
7	If 2.8 <= Strand < 5.29	HET	0.57	0.04	1	0.089	1	1	1	0.670
8	If 17.57 <= Helix < 20.87	ENZ	0.55	0.03	1	0.102	1	1	1	0.668
9	If SCOPClass = 7	ENZ	0.73	0.05	1	0.184	1	1		0.660
10	If 84.76 <= nAtom < 125.14 AND 461.83 <= df-ASA < 681.42	ENZ	0.62	0.05	1	0.145	1	1		0.636
11	If .66 <= inPro < .87	nonENZ	0.6	0.04	1	0.129	1	1		0.628
12	If 205.9 <= nAtom < 246.28	HET	0.57	0.04	1	0.143	1	1		0.626
13	If 12.66 <= sRatio < 15.06 AND 461.83 <= df-ASA < 681.42	ENZ	0.6	0.03	1	0.113	1	1		0.624
14	If 461.83 <= df-ASA < 681.42 AND 10.38 <= nSSE < 12.25 AND SCOPClass = 2	ENZ	0.86	0.03		0.23	1	1		0.624
15	If 26.74 <= nAA < 35.32 AND 901.01 <= df-ASA < 1120.6	nonENZ	0.56	0.03	1	0.133	1	1		0.620
16	If NoHelix AND nFrag < 4.75	ENZ	0.61	0.03		0.076	1	1	1	0.620
17	If 461.83 <= df-ASA < 681.42 AND NoHelix	ENZ	0.59	0.04		0.085	1	1	1	0.619
18	If SCOPClass = 1 AND 1.87 <= LCS < 2.3	nonENZ	0.55	0.03	1	0.137	1	1		0.619
19	If 4.75 <= nSSE < 6.62 AND NoHelix	ENZ	0.59	0.03		0.072	1	1	1	0.615
20	If 1.43 <= LCS < 1.87	nonENZ	0.56	0.04	1	0.074	1	1		0.612
21	If NoStrand AND 1.87 <= LCS < 2.3	nonENZ	0.52	0.04		0.113	1	1	1	0.611
22	If SCOPClass = 1 AND 12.25 <= nFrag < 16	nonENZ	0.79	0.03		0.22	1	1		0.608
23	If 4.75 <= nFrag < 8.5 AND 9.58 <= nAA < 18.16	nonENZ	0.51	0.03	1	0.1	1	1		0.607
24	If NoHelix	ENZ	0.51	0.07		0.058	1	1	1	0.606
25	If 44.91 <= Loop < 48.15	HET	0.48	0.04	1	0.11		1	1	0.604
26	If 67.59 <= Loop < 70.83	ENZ	0.53	0.03		0.048	1	1	1	0.601
27	If SCOPClass = 2 AND 461.83 <= df-ASA < 681.42 AND 84.76 <= nAtom < 125.14	ENZ	0.79	0.03		0.176	1	1		0.599
28	If 461.83 <= df-ASA < 681.42 AND 2.3 <= LCS < 2.73	ENZ	0.63	0.03		0.12	1	1		0.555
29	If 10.38 <= nSSE < 12.25 AND SCOPClass = 2	ENZ	0.58	0.04		0.088	1	1		0.542
30	If 9.58 <= nAA < 18.16 AND 461.83 <= df-ASA < 681.42	ENZ	0.54	0.04		0.058	1	1		0.527
31	If SCOPClass = 3 AND 2.3 <= LCS < 2.73	HOM	0.47	0.03	1	0.1		1		0.521
32	If 3.6 <= LCS < 4.03	HET	0.46	0.04	1	0.1		1		0.520
33	If .44 <= HH < .46	HET	0.47	0.05	1	0.07		1		0.516
34	If 9.58 <= nAA < 18.16 AND 44.38 <= nAtom < 84.76 AND 461.83 <= df-ASA < 681.	ENZ	0.5	0.03		0.045	1	1		0.515
35	If 10.38 <= nSSE < 12.25 AND 12.25 <= nFrag < 16	ENZ	0.5	0.03		0.043	1	1		0.515
36	If 58.11 <= ASAPR < 59.52	nonENZ	0.48	0.03	1	0.065		1		0.515
37	If 57.87 <= Loop < 61.11	ENZ	0.47	0.04		0.045		1	1	0.510
38	If 41.67 <= Loop < 44.91	nonENZ	0.42	0.03		0.046		1	1	0.500
39	If 461.83 <= df-ASA < 681.42	ENZ	0.48	0.11	1	0.076				0.416
40	If SCOPClass = 1 AND NoStrand	nonENZ	0.48	0.06		0.074			1	0.406
41	If 10.38 <= nSSE < 12.25 AND 84.76 <= nAtom < 125.14	ENZ	0.48	0.03		0.054		1		0.391
42	If 84.76 <= nAtom < 125.14 AND 12.25 <= nFrag < 16	ENZ	0.43	0.03		0.094		1		0.389
43	If 12.66 <= sRatio < 15.06	ENZ	0.43	0.05		0.051		1		0.382
44	If 461.83 <= df-ASA < 681.42 AND SCOPClass = 2	ENZ	0.46	0.04		0.03		1		0.382
45	If 2.73 <= LCS < 3.17 AND SCOPClass = 2	ENZ	0.45	0.03		0.029		1		0.377
46	If 125.14 <= nAtom < 165.52 AND 901.01 <= df-ASA < 1120.6	nonENZ	0.41	0.04		0.05		1		0.375
47	If .23 <= inPro < .44	ENZ	0.42	0.04		0.026		1		0.371
48	If .44 <= inPro < .66	ENZ	0.39	0.04	1	0.058				0.371
49	If 461.83 <= df-ASA < 681.42 AND 8.5 <= nFrag < 12.25	ENZ	0.41	0.03		0.041		1		0.371
50	If nFrag < 4.75	ENZ	0.4	0.04		0.025		1		0.368
51	If 461.83 <= df-ASA < 681.42 AND 4.75 <= nSSE < 6.62	ENZ	0.42	0.03		0.018		1		0.367
52	If NoStrand	nonENZ	0.34	0.09		0.035			1	0.365
53	If 84.76 <= nAtom < 125.14 AND 18.16 <= nAA < 26.74	nonENZ	0.4	0.04		0.022		1		0.364
54	If 1120.6 <= df-ASA < 1340.19	ENZ	0.39	0.04		0.018		1		0.363

O	Rule description	type	conf	supp	C	G	T	U	S	I
55	If 9.58 <= nAA < 18.16 AND 44.38 <= nAtom < 84.76	ENZ	0.4	0.03		0.018		1		0.361
56	If 8.5 <= nFrag < 12.25 AND 84.76 <= nAtom < 125.14	nonENZ	0.37	0.03		0.013		1		0.353
57	If 165.52 <= nAtom < 205.9	ENZ	0.37	0.03		0.011		1		0.352
58	If NoStrand	HET	0.3	0.07		0.035			1	0.352
59	If 19.85 <= sRatio < 22.25	HET	0.36	0.04		0.011		1		0.351
60	If 18.16 <= nAA < 26.74 AND 44.38 <= nAtom < 84.76	ENZ	0.36	0.03		0.015		1		0.351
61	If 6.62 <= nSSE < 8.5	ENZ	0.29	0.04	1	0.059				0.349
62	If 461.83 <= df-ASA < 681.42 AND 18.16 <= nAA < 26.74	nonENZ	0.34	0.04		0.017			1	0.349
63	If SCOPClass = 1 AND NoStrand	HET	0.28	0.04		0.074			1	0.348
64	If .42 <= HH < .44	nonENZ	0.35	0.04		0.009		1		0.348
65	If 16 <= nFrag < 19.75	ENZ	0.34	0.04		0.009		1		0.346
66	If .34 <= HH < .36	nonENZ	0.33	0.03		0.009		1		0.343
67	If NoStrand	HOM	0.23	0.06		0.035			1	0.332
68	If NoStrand	ENZ	0.13	0.03		0.035			1	0.299
69	If 1.87 <= LCS < 2.3	nonENZ	0.42	0.09		0.04				0.182
70	If SCOPClass = 1	nonENZ	0.4	0.07		0.067				0.178
71	If 901.01 <= df-ASA < 1120.6	nonENZ	0.41	0.06		0.05				0.176
72	If 2.3 <= LCS < 2.73	ENZ	0.38	0.09		0.032				0.167
73	If 84.76 <= nAtom < 125.14 AND SCOPClass = 2	ENZ	0.41	0.04		0.043				0.164
74	If .44 <= inPro < .66	HET	0.39	0.04		0.058				0.161
75	If 6.62 <= nSSE < 8.5	nonENZ	0.37	0.05		0.059				0.160
76	If 84.76 <= nAtom < 125.14	nonENZ	0.35	0.09		0.038				0.159
77	If 44.38 <= nAtom < 84.76 AND 461.83 <= df-ASA < 681.42	ENZ	0.4	0.06		0.023				0.159
78	If 44.38 <= nAtom < 84.76	ENZ	0.38	0.06		0.022				0.154
79	If SCOPClass = 2	ENZ	0.36	0.09		0.019				0.153
80	If 35.32 <= nAA < 43.9 AND 125.14 <= nAtom < 165.52	nonENZ	0.38	0.04		0.041				0.151
81	If 26.74 <= nAA < 35.32	nonENZ	0.35	0.06		0.041				0.151
82	If 84.76 <= nAtom < 125.14	ENZ	0.33	0.09		0.038				0.151
83	If 125.14 <= nAtom < 165.52	nonENZ	0.35	0.06		0.041				0.150
84	If 35.32 <= nAA < 43.9	ENZ	0.36	0.05		0.035				0.148
85	If 12.25 <= nFrag < 16	nonENZ	0.34	0.06		0.041				0.147
86	If 18.16 <= nAA < 26.74	nonENZ	0.35	0.06		0.019				0.144
87	If SCOPClass = 3	HOM	0.32	0.09		0.016				0.144
88	If 3.17 <= LCS < 3.6	HET	0.36	0.04		0.034				0.143
89	If 8.5 <= nFrag < 12.25	nonENZ	0.35	0.06		0.012				0.141
90	If .4 <= HH < .42	ENZ	0.36	0.04		0.023				0.141
91	If SCOPClass = 1	HET	0.3	0.05		0.067				0.140
92	If 8.5 <= nSSE < 10.38	nonENZ	0.36	0.04		0.017				0.140
93	If 10.38 <= nSSE < 12.25	ENZ	0.36	0.05		0.013				0.139
94	If 84.76 <= nAtom < 125.14 AND 26.74 <= nAA < 35.32	nonENZ	0.35	0.04		0.024				0.139
95	If 4.75 <= nSSE < 6.62	ENZ	0.35	0.04		0.023				0.139
96	If 26.74 <= nAA < 35.32	ENZ	0.32	0.05		0.041				0.138
97	If 125.14 <= nAtom < 165.52	ENZ	0.32	0.05		0.041				0.137
98	If 3.17 <= LCS < 3.6	HOM	0.34	0.04		0.034				0.135
99	If 12.25 <= nFrag < 16	ENZ	0.31	0.05		0.041				0.135
100	If 35.32 <= nAA < 43.9	nonENZ	0.32	0.05		0.035				0.135
101	If 35.32 <= nAA < 43.9 AND 125.14 <= nAtom < 165.52	ENZ	0.32	0.03		0.041				0.132
102	If 6.62 <= nSSE < 8.5	HOM	0.29	0.04		0.059				0.129
103	If 84.76 <= nAtom < 125.14 AND 681.42 <= df-ASA < 901.01	nonENZ	0.34	0.04		0.009				0.129
104	If 461.83 <= df-ASA < 681.42	nonENZ	0.25	0.06		0.076				0.128
105	If 84.76 <= nAtom < 125.14 AND SCOPClass = 2	nonENZ	0.31	0.03		0.043				0.127
106	If 4.75 <= nFrag < 8.5	ENZ	0.31	0.05		0.013				0.126
107	If 4.75 <= nFrag < 8.5	nonENZ	0.31	0.05		0.013				0.126
108	If SCOPClass = 4	HOM	0.31	0.06		0.011				0.126
109	If 681.42 <= df-ASA < 901.01	ENZ	0.32	0.05		0.013				0.126
110	If 681.42 <= df-ASA < 901.01	nonENZ	0.32	0.05		0.013				0.126
111	If 9.58 <= nAA < 18.16	ENZ	0.32	0.04		0.019				0.126
112	If 9.58 <= nAA < 18.16	nonENZ	0.32	0.04		0.019				0.126
113	If 901.01 <= df-ASA < 1120.6	HOM	0.28	0.04		0.05				0.125

O	Rule description	type	conf	supp	C	G	T	U	S	I
114	If 84.76 <= nAtom < 125.14 AND 26.74 <= nAA < 35.32	ENZ	0.31	0.04		0.024				0.123
115	If 4.75 <= nSSE < 6.62	nonENZ	0.31	0.04		0.023				0.122
116	If 18.16 <= nAA < 26.74	ENZ	0.29	0.05		0.019				0.121
117	If .4 <= HH < .42	HOM	0.3	0.04		0.023				0.118
118	If 44.38 <= nAtom < 84.76	nonENZ	0.28	0.05		0.022				0.117
119	If SCOPClass = 2	nonENZ	0.27	0.06		0.019				0.117
120	If 12.25 <= nFrag < 16	HOM	0.26	0.05		0.041				0.116
121	If SCOPClass = 1	HOM	0.24	0.04		0.067				0.116
122	If SCOPClass = 3	HET	0.26	0.07		0.016				0.115
123	If 2.73 <= LCS < 3.17	ENZ	0.29	0.05		0.007				0.114
124	If 461.83 <= df-ASA < 681.42 AND 18.16 <= nAA < 26.74	ENZ	0.29	0.03		0.017				0.113
125	If SCOPClass = 4	nonENZ	0.28	0.05		0.011				0.113
126	If .38 <= HH < .4	HOM	0.3	0.04		0.004				0.113
127	If 2.3 <= LCS < 2.73	HOM	0.24	0.05		0.032				0.110
128	If 2.73 <= LCS < 3.17	HOM	0.28	0.05		0.007				0.110
129	If 2.3 <= LCS < 2.73	nonENZ	0.24	0.05		0.032				0.108
130	If SCOPClass = 3	ENZ	0.24	0.07		0.016				0.108
131	If 125.14 <= nAtom < 165.52	HOM	0.24	0.04		0.041				0.108
132	If 8.5 <= nSSE < 10.38	HOM	0.27	0.03		0.017				0.106
133	If 26.74 <= nAA < 35.32	HOM	0.24	0.04		0.041				0.106
134	If 44.38 <= nAtom < 84.76 AND 461.83 <= df-ASA < 681.42	nonENZ	0.25	0.04		0.023				0.104
135	If 1.87 <= LCS < 2.3	HET	0.22	0.05		0.04				0.102
136	If 2.73 <= LCS < 3.17	nonENZ	0.26	0.04		0.007				0.102
137	If 84.76 <= nAtom < 125.14 AND 681.42 <= df-ASA < 901.01	ENZ	0.25	0.03		0.009				0.098
138	If .38 <= HH < .4	nonENZ	0.25	0.03		0.004				0.097
139	If SCOPClass = 2	HET	0.22	0.05		0.019				0.096
140	If 35.32 <= nAA < 43.9	HOM	0.22	0.03		0.035				0.096
141	If 901.01 <= df-ASA < 1120.6	ENZ	0.21	0.03		0.05				0.096
142	If 8.5 <= nFrag < 12.25	ENZ	0.23	0.04		0.012				0.095
143	If 10.38 <= nSSE < 12.25	nonENZ	0.24	0.03		0.013				0.094
144	If 10.38 <= nSSE < 12.25	HOM	0.24	0.03		0.013				0.094
145	If 1.87 <= LCS < 2.3	HOM	0.2	0.04		0.04				0.093
146	If SCOPClass = 4	ENZ	0.22	0.04		0.011				0.091
147	If 8.5 <= nFrag < 12.25	HOM	0.22	0.04		0.012				0.091
148	If 84.76 <= nAtom < 125.14	HOM	0.18	0.05		0.038				0.087
149	If 18.16 <= nAA < 26.74	HET	0.2	0.04		0.019				0.086
150	If 461.83 <= df-ASA < 681.42	HOM	0.15	0.03		0.076				0.085
151	If SCOPClass = 3	nonENZ	0.18	0.05		0.016				0.084
152	If 8.5 <= nFrag < 12.25	HET	0.2	0.04		0.012				0.084
153	If 4.75 <= nFrag < 8.5	HOM	0.2	0.03		0.013				0.082
154	If SCOPClass = 4	HET	0.19	0.04		0.011				0.080
155	If 1.87 <= LCS < 2.3	ENZ	0.16	0.03		0.04				0.077
156	If 84.76 <= nAtom < 125.14	HET	0.14	0.04		0.038				0.073
157	If SCOPClass = 2	HOM	0.16	0.04		0.019				0.072

Common Rules

Type1, Type2, Type3 and Type4 referred to ENZ, nonENZ, HET and HOM respectively. A list of representatives of overlapping rules between two types is demonstrated as follows:

Common rules for Type 1 and Type 2:

1. If SCOPClass = 2 AND $84.76 \leq$ nAtom $< 125.14 \rightarrow$ Type1 (0.408,0.042) AND Type2 (0.306, 0.032)
2. If $461.83 \leq$ df-ASA < 681.42 AND $44.38 \leq$ nAtom $< 84.76 \rightarrow$ Type1 (0.396, 0.058) AND Type2 (0.252, 0.037)
3. If $4.75 \leq$ nSSE $< 6.62 \rightarrow$ Type1 (0.351, 0.042) AND Type2 (0.307, 0.037)
4. If $35.32 \leq$ nAA < 43.9 AND $125.14 \leq$ nAtom $< 165.52 \rightarrow$ Type1 (0.323, 0.032) AND Type2 (0.376, 0.037)
5. If $681.42 \leq$ df-ASA $< 901.01 \rightarrow$ Type1 (0.317, 0.048) AND Type2 (0.317, 0.048)
6. If $26.74 \leq$ nAA < 35.32 AND $84.76 \leq$ nAtom $< 125.14 \rightarrow$ Type1 (0.307, 0.037) AND Type2 (0.351, 0.042)
7. If $6.62 \leq$ nSSE $< 8.5 \rightarrow$ Type1 (0.294,0.042) AND Type2 (0.368, 0.053)
8. If $461.83 \leq$ df-ASA < 681.42 AND $18.16 \leq$ nAA $< 26.74 \rightarrow$ Type1 (0.291, 0.032) AND Type2 (0.34, 0.037)

Common rules for Type 1 and Type 3:

9. If $0.44 \leq$ inPro $< 0.66 \rightarrow$ Type1 (0.389, 0.037) AND Type3(0.389,0.037)

Common rule for Type 1 and Type 4:

1. If $0.4 \leq$ HH $< 0.42 \rightarrow$ Type1 (0.357, 0.042) AND Type4 (0.295, 0.035)
2. If $6.62 \leq$ nSSE $< 8.5 \rightarrow$ Type1 (0.294, 0.042) AND Type4 (0.287,

Common rule for Type 2 and Type 3:

1. If SCOPClass = 1 AND Nostrand $\rightarrow$ Type2 (0.484, 0.064) AND Type3(0.282, 0.037)

Common rule for Type 2 and Type 4:

1. If $901.01 \leq$ df-ASA $< 1120.6 \rightarrow$ Type2 (0.414, 0.064) AND Type4(0.283, 0.043)
2. If $6.62 \leq$ nSSE $< 8.5 \rightarrow$ Type2 (0.368,0.053) AND Type4 (0.287,0.041)

Common rule for Type 3 and Type 4:

1. If $3.17 \leq$ LCS $< 3.6 \rightarrow$ Type3(0.357, 0.037) AND Type4 (0.337,0.035)
2. If $0.42 \leq$ HH $< 0.48 \rightarrow$ Type 3(0.313, 0.031) AND Type 4(0.352, 0.048)
